# Silicon nanoparticles (SiNPs) restore photosynthesis and essential oil content by upgrading enzymatic antioxidant metabolism in lemongrass (*Cymbopogon flexuosus*) under salt stress

**DOI:** 10.3389/fpls.2023.1116769

**Published:** 2023-02-17

**Authors:** Mohammad Mukarram, M. Masroor A. Khan, Daniel Kurjak, Alexander Lux, Francisco J. Corpas

**Affiliations:** ^1^ Advance Plant Physiology Section, Department of Botany, Aligarh Muslim University, Aligarh, India; ^2^ Department of Phytology, Faculty of Forestry, Technical University in Zvolen, Zvolen, Slovakia; ^3^ Department of Integrated Forest and Landscape Protection, Faculty of Forestry, Technical University in Zvolen, Zvolen, Slovakia; ^4^ Department of Plant Physiology, Faculty of Natural Sciences, Comenius University in Bratislava, Ilkovicova 6, Bratislava, Slovakia; ^5^ Institute of Chemistry, Slovak Academy of Sciences, Bratislava, Slovakia; ^6^ Department of Stress, Development and Signaling in Plants, Antioxidant, Free Radical and Nitric Oxide in Biotechnology, Food and Agriculture Group, Estación Experimental del Zaidín, Consejo Superior de Investigaciones Científicas (CSIC), Granada, Spain

**Keywords:** nanoparticles, antioxidants, oxidative stress, photosynthesis, reactive oxygen species, salinity, silica, medicinal plant

## Abstract

Lemongrass (*Cymbopogon flexuosus*) has great relevance considering the substantial commercial potential of its essential oil. Nevertheless, the increasing soil salinity poses an imminent threat to lemongrass cultivation given its moderate salt-sensitivity. For this, we used silicon nanoparticles (SiNPs) to stimulate salt tolerance in lemongrass considering SiNPs special relevance to stress settings. Five foliar sprays of SiNPs 150 mg L^-1^ were applied weekly to NaCl 160 and 240 mM-stressed plants. The data indicated that SiNPs minimised oxidative stress markers (lipid peroxidation, H_2_O_2_ content) while triggering a general activation of growth, photosynthetic performance, enzymatic antioxidant system including superoxide dismutase (SOD), catalase (CAT), and peroxidase (POD), and osmolyte proline (PRO). SiNPs amplified stomatal conductance and photosynthetic CO_2_ assimilation rate by about 24% and 21% in NaCl 160 mM-stressed plants. Associated benefits contributed to pronounced plant phenotype over their stressed counterparts, as we found. Foliar SiNPs sprays assuaged plant height by 30% and 64%, dry weight by 31% and 59%, and leaf area by 31% and 50% under NaCl 160 and 240 mM concentrations, respectively. SiNPs relieved enzymatic antioxidants (SOD, CAT, POD) and osmolyte (PRO) in lemongrass plants stressed with NaCl 160 mM (9%, 11%, 9%, and 12%, respectively) and NaCl 240 mM (13%, 18%, 15%, and 23%, respectively). The same treatment supported the oil biosynthesis improving essential oil content by 22% and 44% during 160 and 240 mM salt stress, respectively. We found SiNPs can completely overcome NaCl 160 mM stress while significantly palliating NaCl 240 mM stress. Thus, we propose that SiNPs can be a useful biotechnological tool to palliate salinity stress in lemongrass and related crops.

## 1 Introduction

Salt-induced damage to growth and productivity depends on the plant tolerance to salt which varies significantly among species, and since most plants are glycophytes, increasing salinity poses an imminent threat to global agriculture and food security (Munns and Tester, 2008; Butcher et al., 2016). Higher salt concentrations impede plant water uptake creating osmotic stress in a drought-like manner (Munns, 2002). The osmotic stress instigates ion excess and disrupts ion homeostasis staging ionic stress (Van Zelm et al., 2020). The physiological setbacks are further exacerbated by oxidative stress. At this point, the plant has the excessive presence of hydrogen peroxide (H_2_O_2_), superoxide anion ( ${\text{O}}_{2}^{\cdot -}$ ), singlet oxygen (^1^O_2_), and hydrogen radical (^•^OH) in the chloroplast, mitochondria, peroxisomes, endoplasmic reticulum, vacuole, cytoplasm, and apoplast (Foyer, 2018; Corpas et al., 2020; Mittler et al., 2022). These oxidative species are collectively known as reactive oxygen species (ROS). ROS degenerate cellular integrity through oxidising lipid bilayer and destabilising the structure and function of the proteins, lipids, and nucleic acids (Miller et al., 2010; Hossain et al., 2017). Most of the cellular organelles comprise a set of specialised compounds capable of scavenging ROS. These compounds, antioxidants, lay the first line of defence against mounting oxidative stress. Therefore, a robust antioxidative system can be indispensable in determining plant stress tolerance (Foyer and Noctor, 2005; Foyer and Shigeoka, 2011; Hasanuzzaman et al., 2020). Further, several attempts to advance crop performance under salt stress manifest a strong correlation with the upregulated antioxidant system (Hernandez et al., 2000; Zhu et al., 2004; Ashraf, 2009; Houmani et al., 2016; Ashraf and Munns, 2022).

Agricultural intensifications to boost crop productivity coupled with climate change and poor agricultural practices such as the excessive use of traditional fertilisers have further worsened the soil biochemical texture and exaggerated the saline influence in the soil (Foucher et al., 2014; Kopittke et al., 2019; Eswar et al., 2021; Hopmans et al., 2021). Thus, recent interventions engage sustainable, non-toxic, economic, and environment-friendly alternatives for yield enhancement during optimal and stressful conditions (Lehmann, 2007; Dodd and Pérez-Alfocea, 2012; Machado and Serralheiro, 2017; Mukarram et al., 2022a). The use of silicon nanoparticles (SiNPs) is one such recent intervention. On many occasions, the growth-promoting potential of SiNPs during both optimal and stressful environments was reported (Tripathi et al., 2015; Nazaralian et al., 2017; Fatemi et al., 2021; González-Moscoso et al., 2022). SiNPs, smaller than bulk silicon, might be absorbed faster and provide a higher surface area to interact with plant signalling molecules (Mukarram et al., 2022b). It renders a better plant performance with SiNPs over bulk silicon during stress. SiNPs can be understood as ‘sponges’ that can chelate mineral nutrients and hold moisture for plant roots (Mukarram et al., 2022b). SiNPs can further induce secondary and lateral root growth which might also reinforce plant water and nutrient uptake (El-Dengawy et al., 2021). This enables SiNPs to have special relevance in salinity studies worldwide. Additionally, silica deposition in the apoplast may provide structural strength and better leaf posture for maximised photon flux (Tamai and Ma, 2008). This can improve canopy photosynthesis.

Another moiety of SiNPs action is its active participation in ROS and antioxidant metabolism during salt stress. Several studies suggest upregulated activities of superoxide dismutase (SOD), catalase (CAT), and peroxidase (POD) enzymes with SiNPs (Tripathi et al., 2015; Elsheery et al., 2020; Fatemi et al., 2020; El-Saadony et al., 2021; Mukarram et al., 2021a). This can correspond to silicon’s potential to upregulate the expression level of several genes encoding these antioxidants (Khandekar and Leisner, 2011; Ma et al., 2016; Farouk et al., 2020; Lesharadevi et al., 2021). SiNPs can also have a significant role in osmotic adjustment by regulating the expression of genes connected to proline (PRO) biosynthesis. On many occasions, SiNPs appeased PRO content and supported total antioxidant capacity in salt-stressed plants (Abdel-Haliem et al., 2017; Kalteh et al., 2018; Larkunthod et al., 2022; Mahmoud et al., 2022). Thus, SiNPs-induced structural and physiological modifications, in concert, can ascribe to higher plant growth, development, and productivity under saline scenarios.

A significant part of agricultural land in India is affected by soil salinity curbing gross national crop productivity (Kumar and Sharma, 2020). While the salt implications were extensively studied in many leading crops of the nation, lemongrass received little attention even when India is one of the largest producers and exporters of lemongrass and its essential oil (Skaria et al., 2006). The primary incentive for lemongrass production is its immense commercial potential in the pharmaceutical, food packaging, and cosmetic industries (Haque et al., 2018). Lemongrass export in India has swollen by >1250% in the past two decades, and the future market could be expected to grow (Mukarram et al., 2021b). However, we recently found that lemongrass is moderately salt-sensitive and higher saline concentrations (>NaCl 80 mM) significantly reduce its essential oil productivity (Mukarram et al., 2022c). Therefore, corrective measures are required to shield the lemongrass oil market from growing salinity. In our previous study, SiNPs did not bring enormous defence elicitation in unstressed lemongrass (Mukarram et al., 2021a). However, considering the special relevance of SiNPs under stress, it would be interesting to quantify the responses of the ROS and antioxidative system in salt-stressed (NaCl 160 and 240 mM) lemongrass. Thus, the working hypothesis for this study was that SiNPs cushion lemongrass against salt stress and furnish increased growth and yield (H1). Further, the cornerstone of this tolerance is SiNPs-upgraded ROS and antioxidative system that corresponds to cellular homeostasis in lemongrass during salt excess (H2).

## 2 Material and methods

### 2.1 Plant material and growth condition

Lemongrass (*Cymbopogon flexuosus* (Steud.) Wats) var. Nima developed by the Central Institute of Medicinal and Aromatic Plants, Lucknow, India, was used as plant material for this study. After surface sterilisation with 0.2% HgCl_2_ for 5 min, slips were washed repetitively with deionised water. Plant slips were transferred to soil-filled earthen pots (25 × 25 cm) in a net house at the Department of Botany, Aligarh Muslim University (AMU), Aligarh (27°52' N, 78°51' E, and 187 m a.s.l.). The ranges for temperature (27–36 ± 4°C) and relative humidity ranges (68–74 ± 7%) were recorded for the experimental timeline. Soil samples were randomly collected from different pots for soil analyses at the Soil-Testing Laboratory, Indian Agricultural Research Institute, New Delhi. The soil texture was identified as sandy loam while other variables were as follows: pH (1:2): 7.6, E.C. (1:2): 0.52 m mhos cm^−1^, available N, P and K: 94.8, 8.9, and 136.5 mg kg^-1^ of soil, respectively.

### 2.2 Salinity treatment

Lemongrass plants were maintained under two NaCl concentrations (160 and 240 mM). These concentrations were selected as per our earlier finding on the salt sensitivity of lemongrass plants (Mukarram et al., 2022c). Salt treatments began 21 days after transplantation. NaCl concentrations were supplied as 300 mL of 40 mM NaCl solutions every alternate day to attain the final concentration and to avoid osmotic shock. The control group was supplied only with 300 mL of double distilled water.

### 2.3 Acquisition, structural analysis, and application of silicon nanoparticles

**Figure 1:**
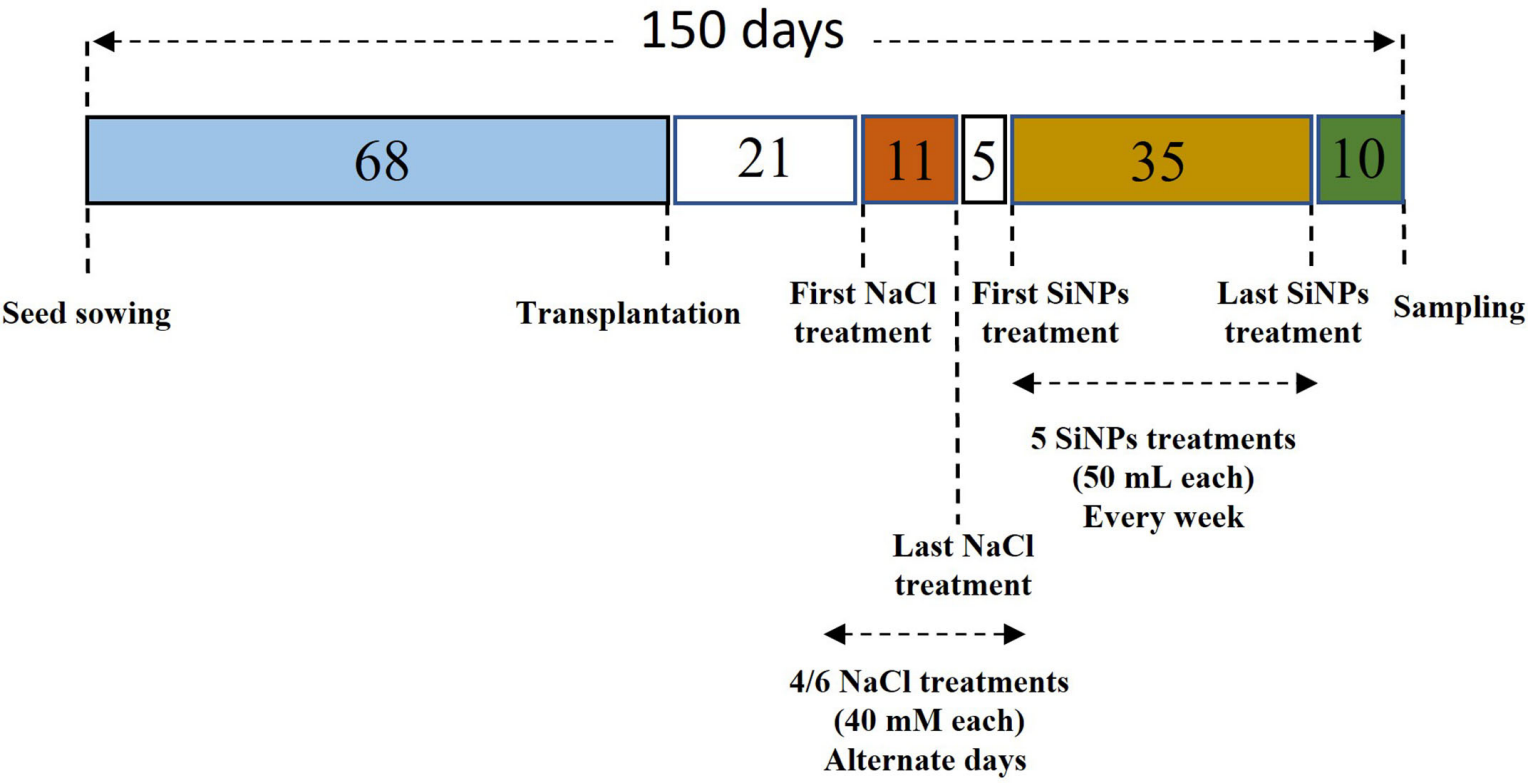
The experimental timeline of the significant events during the present study.

SiNPs were obtained as fumed silica from Evonic Industries AG, Germany, in the form of Aerosil R812. The structural analysis of SiNPs was performed by scanning electron microscopy (JOEL, JSM-6510 LV, Japan) at the Ultra Sophisticated Instrumentation Facility, AMU, Aligarh. SiNPs R812 samples were mounted on the holder with carbon tape and gold was used as a coating material for the scanning. An accelerating current of 10 kilovolts was passed and SEM was set at 1,000× magnification with a spot size of 40 to reveal the nanoparticles structural characteristics. Photos were taken at 10 μm using a secondary electron imaging detector. SiNPs were dissolved using 30% ethyl alcohol and de-ionised water to make a concentration of 150 mg L^-1^. In total, five foliar sprays of SiNPs (50 mL each) were applied every week, starting 5 days after the attainment of the final salt concentration for each group. The schedule in **Figure 1** illustrates the experimental timeline and the protocol employed for NaCl and SiNPs treatments.

### 2.4 Determination of photosynthesis and gas-exchange modules

A saturation-pulse fluorometer PAM-2000 (Walz, Effeltrich, Germany) was used to evaluate chlorophyll fluorescence (Fv/Fm). The plants were dark-adapted for 30 minbefore measuring fluorescence. We recorded Fv/Fm during daytime in the upper surface of the first stretched-out leaf. Chlorophyll content was quantified in the intact expanded leaves of lemongrass through a Minolta chlorophyll meter (SPAD-502; Konica Minolta Sensing Inc. Japan).

Photosynthetic CO_2_ assimilation rate (*A*), stomatal conductance (g_s_), and transpiration rate (E) of the first fully expanded leaf of lemongrass were determined with an infra-red gas analyzer (LiCOR 6200, Portable Photosynthesis System, NA, USA). All the modules were determined on a leaf area of 6 cm^2^ with air temperature, relative humidity, and atmospheric CO_2_ concentration maintained at 25°C, 85%, and 400 ± 5 μmol mol^–1^, respectively. All the measurements were made between 09:00 and 12:00 h at a photosynthetic photon flux density of 780–800 μmol m^-2^ s^-1^.

### 2.5 H_2_O2 content and lipid peroxidation assays

Hydrogen peroxide (H_2_O_2_) content was determined by a peroxidase-dependent assay adopting the method of Okuda et al. (1991). The reaction solution was prepared with crude extract (1 mL), 3- (dimethylamino) benzoic acid (12.5 mM, 0.4 mL), phosphate buffer (37.5 mM, pH 6.5), 3- methyl-2-benzothiazoline hydrazone (0.08 mL). Subsequently, peroxidase (0.02 mL, 0.25 unit) was added as a reaction initiator to the final volume of 1.5 mL at 25°C. The degree of light absorbance was observed for 3 min through a spectrophotometer adjusted at 590 nm.

The thiobarbituric acid reactive substances (TBARS) content was used to signify lipid peroxidation in lemongrass leaves as per Cakmak and Horst (1991). The TBARS concentration was captured as malondialdehyde (MDA) equivalents. For this, leaf tissue (0.5 g) was ground with trichloroacetic acid (0.1% (w/v), 5 ml) with subsequent centrifugation (12,000× g, 5 min). Tetrabutylammonium (0.5% (w/v), 4 mL) in trichloroacetic acid was added to the supernatant followed by incubation and centrifugation. The absorbance was noted at 532 nm and corrected for non-specific turbidity by subtracting the optical density at 600 nm.

### 2.6 Plant crude extracts

For the enzymatic assays, 0.5 g of fresh and mature lemongrass leaves were ground in liquid N_2_ using a mortar and pestle. The resulting coarse powder was transferred to 5 ml (w/v) of chilled extraction medium containing potassium phosphate buffer (100 mM, pH 7.8), 1% (w/v) polyvinylpyrrolidone and 0.5% (v/v) Triton-X-100. Homogenates were centrifuged at 15,000× g for 5 min at 4°C. The supernatant acquired after centrifugation was used to determine enzymatic antioxidant activities (Kuo et al., 1982).

### 2.7 Enzymatic activity assays

The superoxide dismutase (SOD, E.C. 1.15.1.1) activity was assayed according to Beyer and Fridovich (1987). Riboflavin (1 mM), methionine (9.9 mM), nitrobluetetrazolium (55 mM), EDTA (2 mM), and Triton-X-100 (0.02%) were added to the 0.1 mL of freshly prepared enzyme extract and illuminated and maintained for one hour at 30°C. The reaction mixture was analysed by a spectrophotometer (Shimadzu UV-1700, Tokyo, Japan), and absorbance was recorded at 560 nm. One SOD unit is the amount of the enzyme needed for half inhibition of nitrobluetetrazolium reaction at the set wavelength.

The activity of the catalase (CAT, E.C. 1.11.1.6) was determined with the methods of Beers and Sizer (1952) with slight modification. In the 0.04 mL of the leaf extract, 2.6 mL of potassium phosphate buffer (50 mM with pH 7) was added. The solution was centrifuged afterwards at 12,500× g for 20 min at 4°C. The supernatant was removed and 0.4 mL of H_2_O_2_ (15 mM) was added as the reaction substrate. The enzyme activity was measured by determining the disappearance of H_2_O_2_ at 240 nm for 2 minutes with 5 seconds intervals.

The peroxidase (POD, EC 1.11.1.7) activity was estimated by pyrogallol oxidation according to Kumar and Khan (1982). The reaction mixture contained phosphate buffer (0.1 M, pH 6.8, 2 mL), pyrogallol (0.01 M, 1 mL), crude extract (0.5 mL), and H_2_O_2_ (0.005 M, 1 mL). After the incubation (5 min, 25°C), the reaction was stopped with H_2_SO_4_ (2.5 N, 1 mL). The purpurogallin formed by pyrogallol oxidation was measured at 420 nm against a reagent blank.

For geraniol dehydrogenase (GeDH, EC 1.1.1.183) activity, the fresh young leaves (0.5 g) were homogenised into Tricine-NaOH (50 mM, pH 7.5), β-mercaptoethanol (2.5 mM), thiourea (5 mM), phenylmethylsulfonylfluoride (1 mM), and glycerol (15% v/v) in the presence of polyvinylpolypyrrolidone (Polyclar AT) and amberlite XAD-4 as described in the earlier study (Sangwan et al., 1993). Enzyme activity was calculated by determining geraniol-dependent-NADP^+^ reduction and recording absorbance increment at 340 nm.

### 2.8 Proline (PRO) content

The proline was detected in lemongrass leaves according to Bates et al. (1973). The absorbance of the toluene-aspired layer was noted at 520 nm by a spectrophotometer (Shimadzu UV-1700, Tokyo, Japan).

### 2.9 Estimation of growth modules

Growth parameters were evaluated in terms of plant height, dry weight, and leaf area. The lemongrass plants were left to dry for 24 h at 80°C in a hot-air oven to acquire dry weights measurement. Further, the leaf area was determined by following the procedure of Pandey and Singh (2011).

### 2.10 Extraction of lemongrass essential oil

The lemongrass essential oil was acquired by the hydro-distillation method (Guenther, 1972).

### 2.11 Statistical analyses

The experiment was set up in a simple randomised design. At least five independent biological replicas were used for each treatment and evaluation of various parameters. The normal distribution of the data was first tested for each treatment by the Shapiro–Wilk test. The homogeneity of variance among treatments was tested with Bartlett’s test. One-way analysis of variance (ANOVA) was used to test the SiNPs effect on lemongrass growth, development, and productivity under salinity stress. Duncan’s mean range *post-hoc* test was used to determine the significance of differences among the treatments. All statistical analyses were conducted at the replicate level and α = 0.05 in RStudio (RStudio PBC, Boston, MA, USA). Principal component analysis (PCA) was applied to measured parameters using FactoMineR and factoextra packages to explore the positioning of each treatment with the remaining treatment groups. Further, the relationship among studied parameters was drawn using the PerformanceAnalytics package and presented in the correlation matrix. Correlation analysis was used to analyse relationships among all parameters observed for control and treated plants.

## 3 Results

### 3.1 SiNPs characterisation

**Figure 2:**
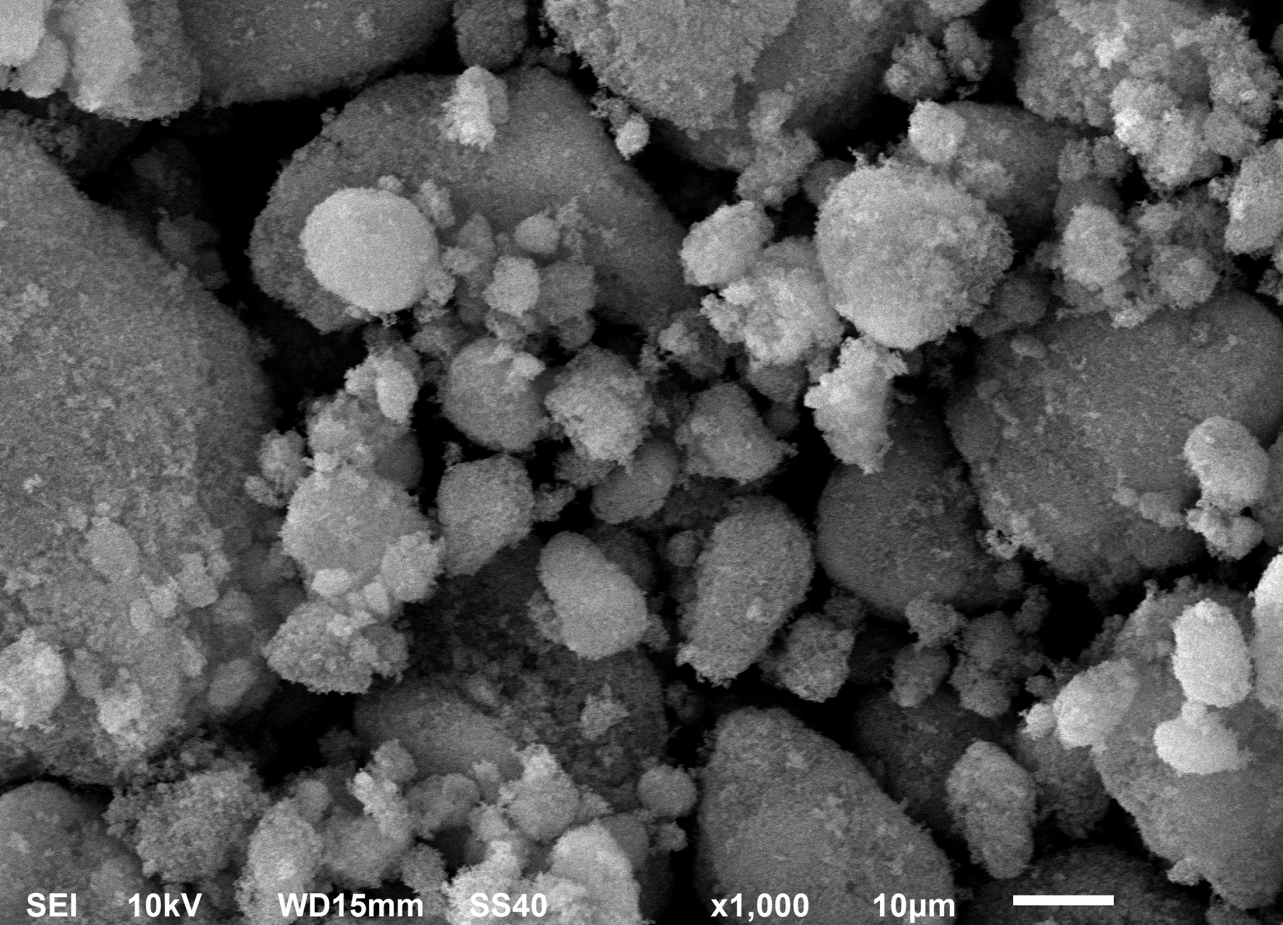
Scanning electron microscopy (SEM) representative picture of silicon nanoparticles (SiNPs) used in this study. The average size for R812 SiNPs was calculated as 7 nm with a specific surface area of 200 m^2^ g^-1^.

The average particle diameter of R812 was 7 nm with a specific surface area of 200 m^2^ g^-1^ (**Figure 2**). R812 is a hydrophobic derivative of Aerosil 300 which is composed of untreated fumed silica powder with a BET surface area of 300 m^2^ g^-1^. Aerosil 300 are reacted with hexamethyldisilazane to form methylated and hydrophobic R812 nanoparticles.

### 3.2 SiNPs appease salinity-induced growth constraints in lemongrass

**Figure 3:**
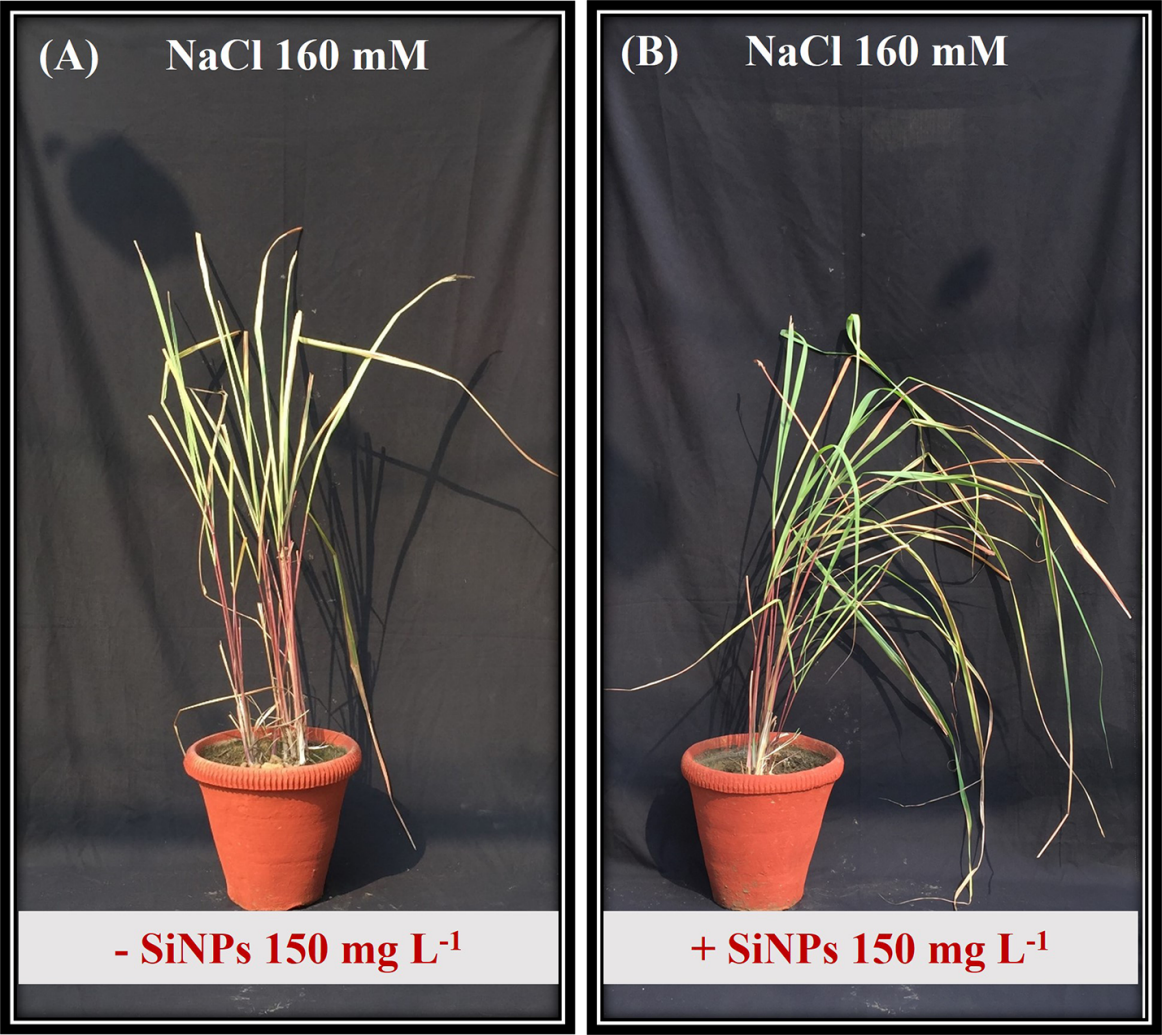
Phenotype of lemongrass plant under NaCl 160 mM salinity regime without **(A)** and with **(B)** SiNPs application (150 mg L^-1^).

**Figure 4:**
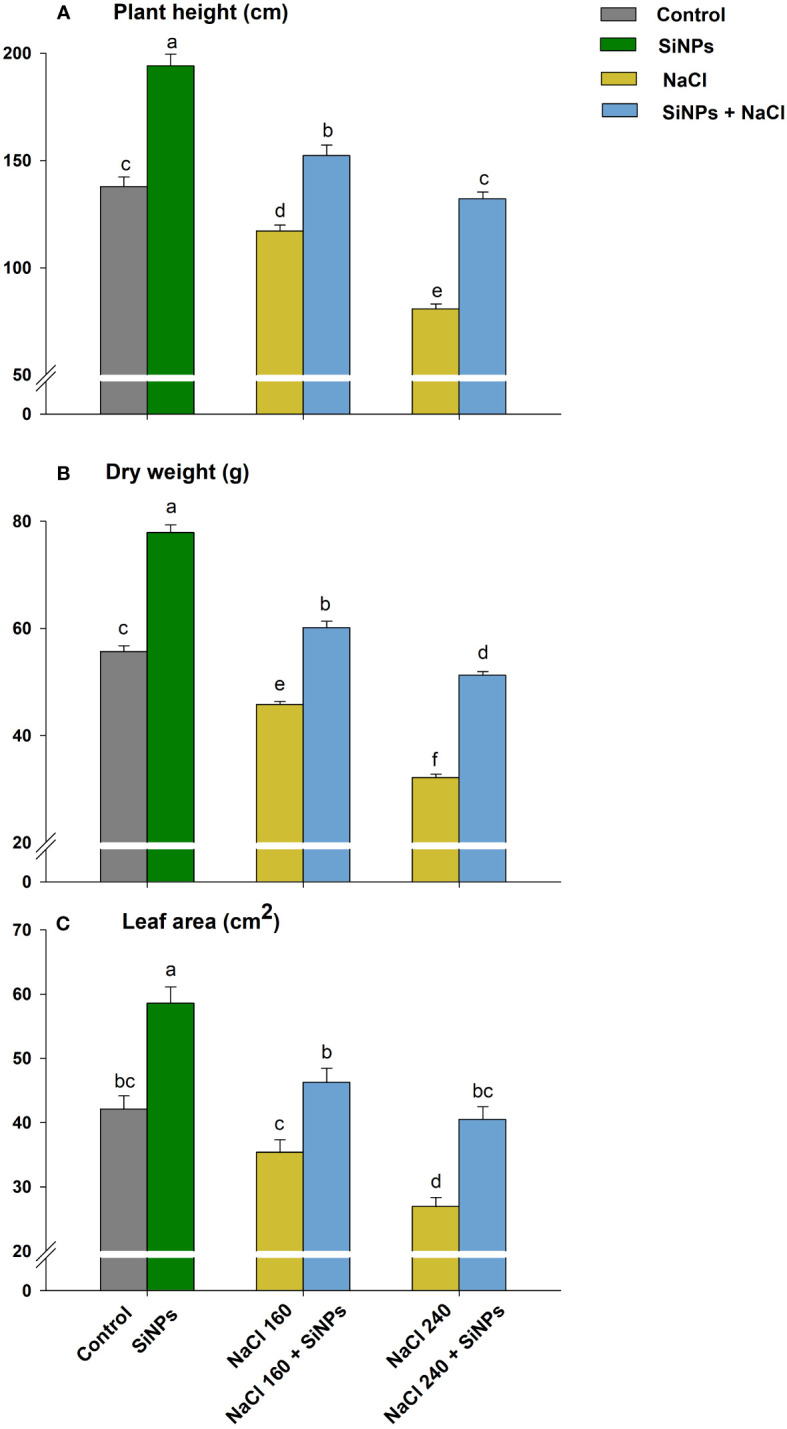
Effect of SiNPs on lemongrass plant height **(A)**, dry weight **(B)**, and leaf area **(C)** under salt stress. Replicate mean ± standard deviation is represented for each value. The difference between the mean values having the same letter(s) within a column is insignificant (α ≤ 0.05) according to Tukey’s HSD test. The concentrations are expressed in mM (NaCl) and mg L^-1^ (SiNPs).

The visible effect of salt stress comprised redundant growth, shorter plants, and fewer green leaves (**Figure 3**). The saline regime reduced plant height and dry weight under both concentrations (NaCl 160 & 240 mM) (**Figures 4A, B**). Leaf expansion faced a similar deleterious impact with the highest reduction in leaf area being at NaCl 240 mM (**Figure 4C**). Nonetheless, when salt-stressed plants were treated with SiNPs, they acquired improved growth. Under NaCl 160 mM treatments, SiNPs sprays improved plant height, dry weight, and leaf area each by about 30% while during NaCl 240 mM stress, it was 64%, 59%, and 50%, respectively. Although SiNPs could restore plant performance significantly under NaCl 240 mM, the complete salinity reversal was observed under NaCl 160 mM.

### 3.3 SiNPs upgrade photosynthesis and stomatal activity under salinity

**Figure 5:**
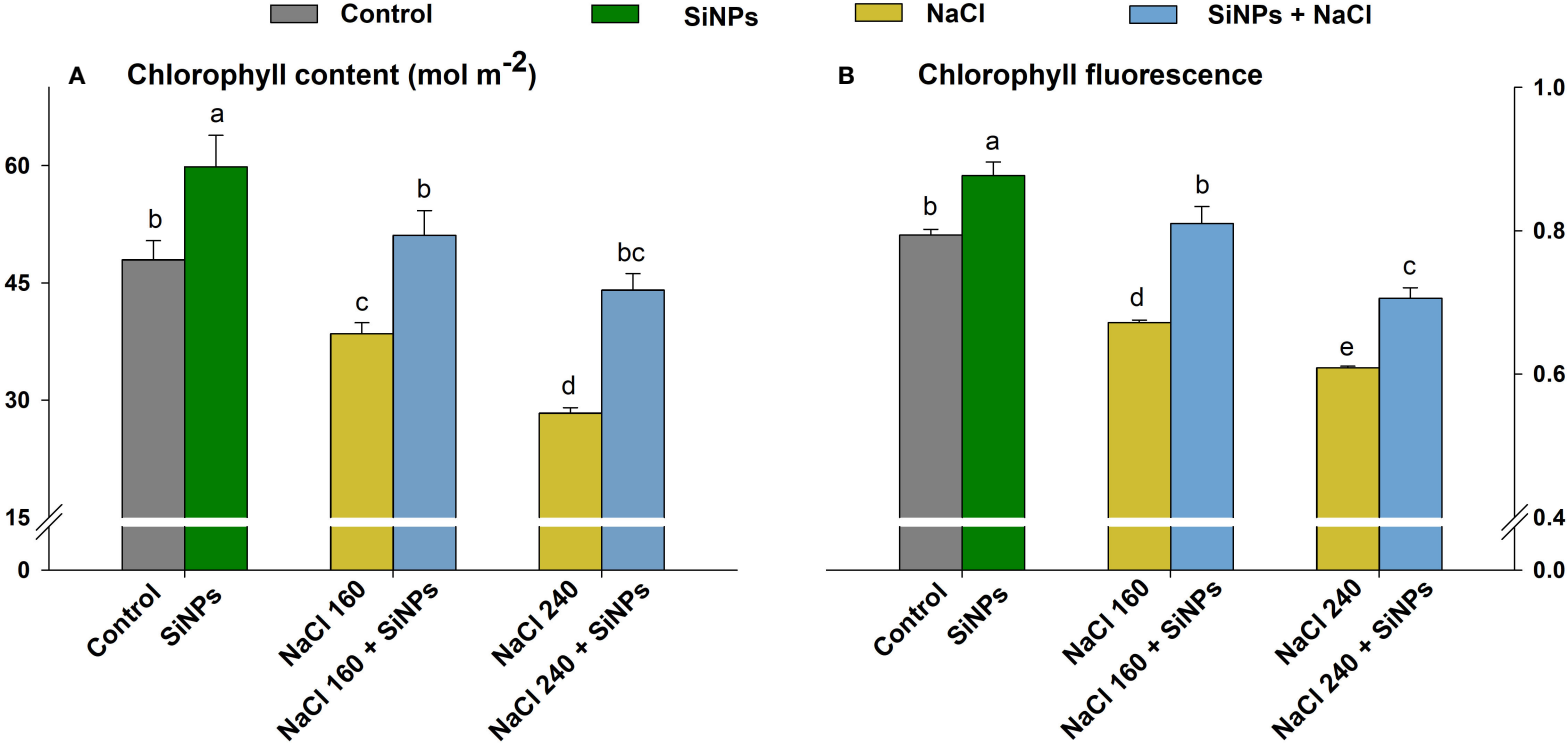
SiNPs effect on chlorophyll content **(A)** and chlorophyll fluorescence (Fv/Fm) **(B)** of lemongrass under salinity. Replicate mean ± standard deviation is represented for each value. The difference between the mean values having the same letter(s) within a column is insignificant (α ≤ 0.05) according to Tukey’s HSD test. The concentrations are expressed in mM (NaCl) and mg L^-1^ (SiNPs).

Lemongrass plants grown under salt concentrations (160 & 240 mM) displayed minimised photosynthesis. However, the foliar sprays with SiNPs 150 mg L^-1^ improved photosynthetic traits under the physiological and saline domains. The experimental results suggest that the extreme salinity (NaCl 240 mM) minimised chlorophyll biosynthesis. However, SiNPs brought the highest salt amelioration in such plants with improved chlorophyll content (55%) and fluorescence (Fv/Fm) (16%) (**Figures 5A, B**).

**Figure 6:**
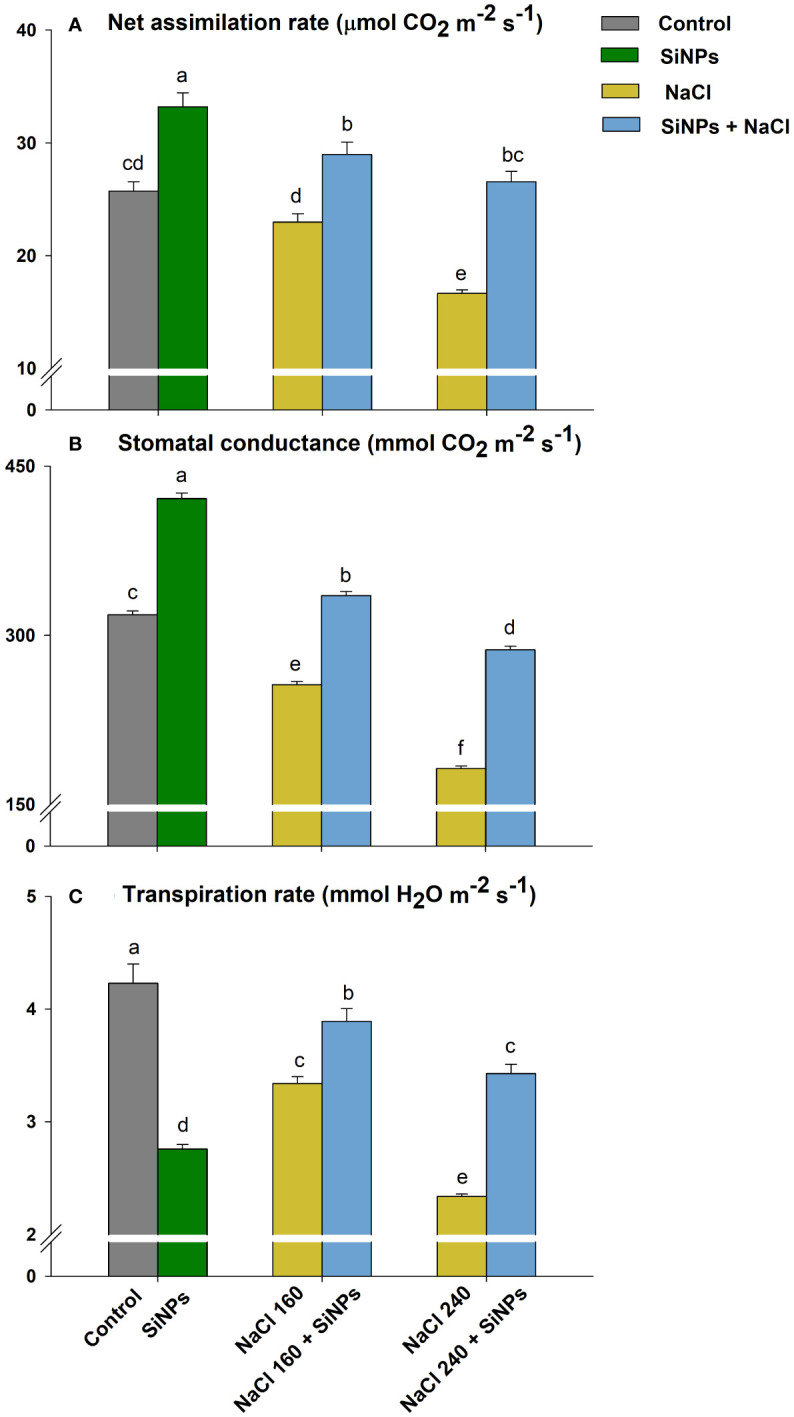
SiNPs effect on stomatal dynamics such as net CO_2_ assimilation rate **(A)**, stomatal conductance **(B)**, and transpiration rate **(C)** of lemongrass under salinity. Replicate mean ± standard deviation is represented for each value. The difference between the mean values having the same letter(s) within a column is insignificant (α ≤ 0.05) according to Tukey’s HSD test. The concentrations are expressed in mM (NaCl) and mg L^-1^ (SiNPs).

Salinity also negatively influenced stomatal dynamics including *A*, g_s_, and E. The maximum reduction in *A*, g_s_, and E was observed with NaCl 240 mM followed by NaCl 160 mM. Foliar sprays of SiNPs on lemongrass plants growing under salt conditions improved stomatal opening, transpiration, and CO_2_ assimilation. SiNPs treatment to 160 mM NaCl-stressed plants increased E by 16%, g_s_ by 31%, and *A* by 26% over SiNPs-untreated plants (**Figure 6**).

### 3.4 SiNPs bolster ROS metabolism for enhanced salt tolerance

**Figure 7:**
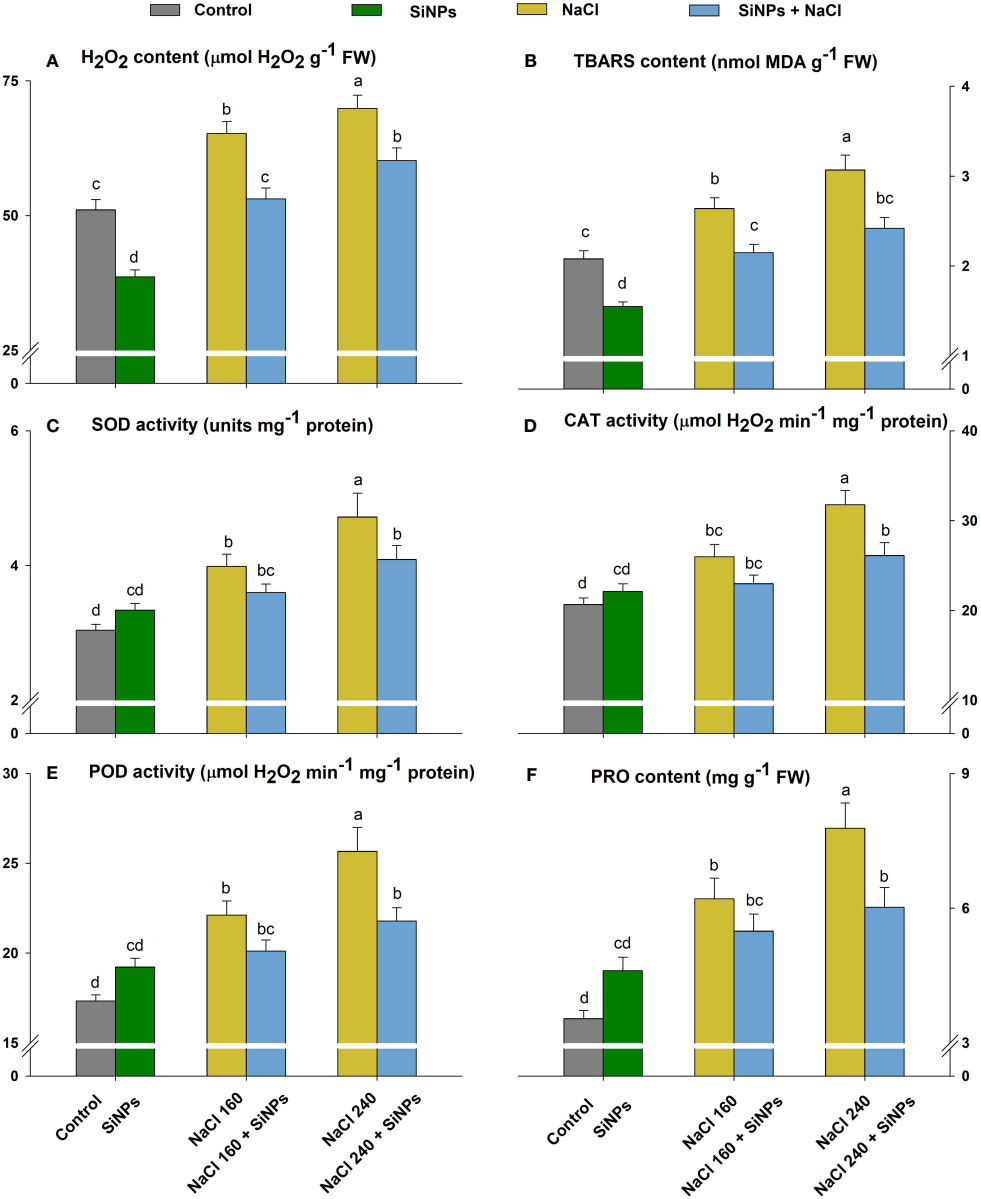
Lemongrass ROS metabolism under salinity as influenced by SiNPs. The antioxidant and ROS metabolism was represented in terms of hydrogen peroxide (H_2_O_2_) content **(A)**, thiobarbituric acid reactive substances (TBARS) content **(B)**, superoxide dismutase (SOD) activity **(C)**, catalase (CAT) activity **(D)**, peroxidase (POD) activity **(E)**, and proline (PRO) content **(F)**. Replicate mean ± standard deviation is represented for each value. The difference between the mean values having the same letter(s) within a column is insignificant (a ≤ 0.05) according to Tukey’s HSD test. The concentrations are expressed in mM (NaCl) and mg L^-1^ (SiNPs).

The ROS metabolism was upregulated to combat rising oxidative stress in lemongrass caused by salinity. Both salt concentrations (160 and 240 mM) increased oxidative stress as depicted by enhanced H_2_O_2_ (28% and 37%) and TBARS (27% and 48%) contents over the control (**Figures 7A, B**). The lemongrass defence system was strengthened by overproduced antioxidative enzymes SOD, CAT, and POD and osmolyte PRO to minimise cellular damage. SiNPs supported antioxidant and osmolyte production in the absence of salinity which implies a positive SiNPs influence on redox homeostasis. The same SiNPs treatment (150 mg L^-1^) appeased H_2_O_2_ contents by 19% and 14% and TBARS contents by 17% and 21% in 160 and 240 mM salt-stressed plants, respectively. Furthermore, SiNPs relieved SOD (9% and 13%), CAT (11% and 18%), and POD (9% and 15%) activities in 160 and 240 mM NaCl-treated plants to achieve a healthier cellular environment (**Figures 7C–E**). SiNPs also assuaged PRO intensification in 160 (12%) and 240 mM (23%) salt-stressed lemongrass plants suggesting a positive role in restricting salinity-induced osmotic stress (**Figure 7F**).

### 3.5 SiNPs appease lemongrass productivity during the saline regime

**Figure 8:**
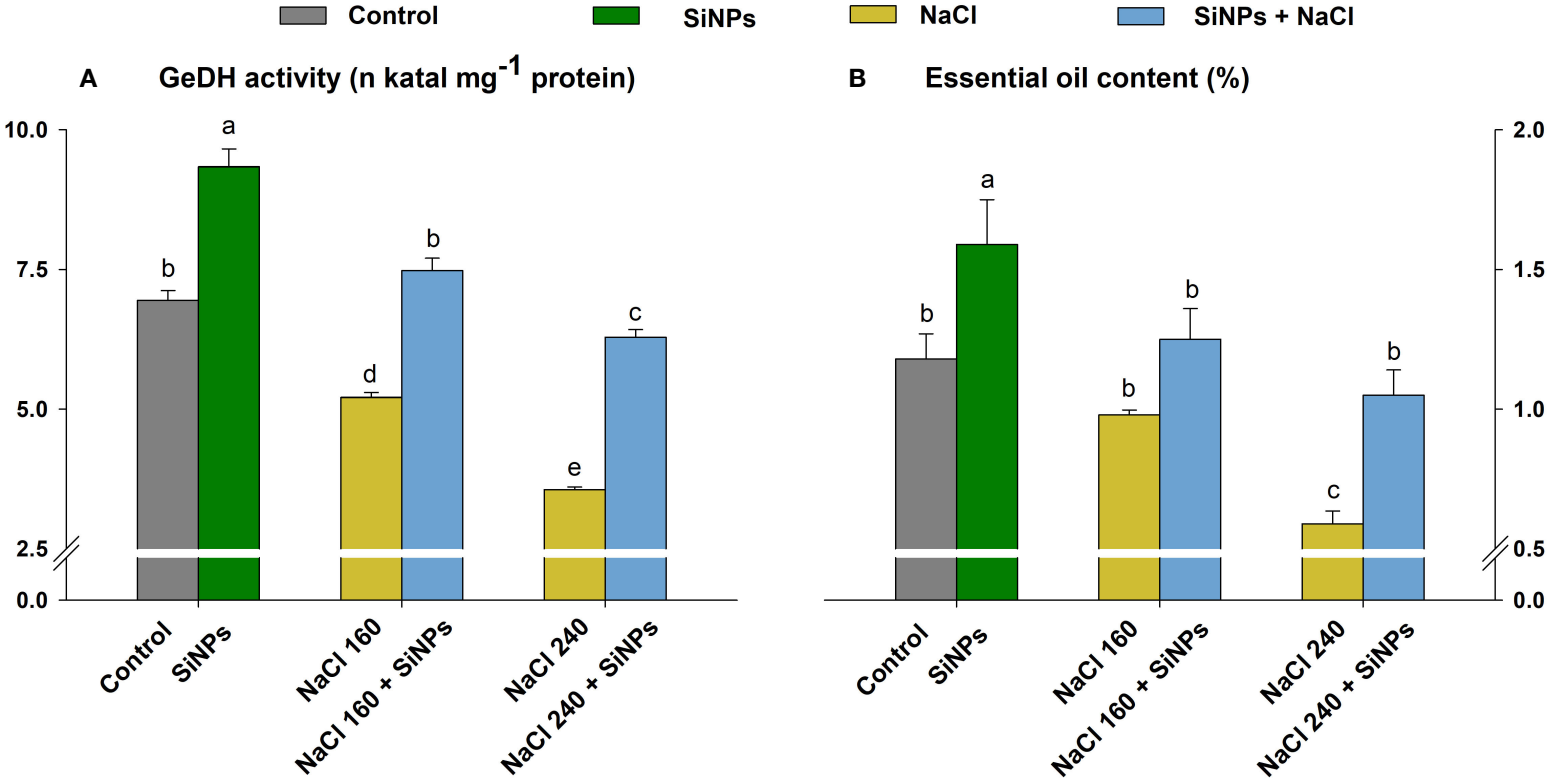
Effect of SiNPs sprays on geraniol dehydrogenase (GeDH) activity **(A)** and essential oil content as a percentage to plant dry weight **(B)** in lemongrass leaves during salinity stress. Replicate mean ± standard deviation is represented for each value. The difference between the mean values having the same letter(s) within a column is insignificant (α ≤ 0.05) according to Tukey’s HSD test. The concentrations are expressed in mM (NaCl) and mg L^-1^ (SiNPs).

Salinity adversely affected lemongrass oil productivity and the enzyme responsible for its production. The maximised reduction in GeDH activity (48%) and essential oil content (50%) was noted with the NaCl 240 mg L^-1^. Nevertheless, SiNPs 150 mg L^-1^ sprays brought significant (α = 0.05) improvement in GeDH activity (43% and 76%) and essential oil content (27% and 78%) during both saline regimes (NaCl 160 and 240 mM) (**Figure 8**).

**Figure 9:**
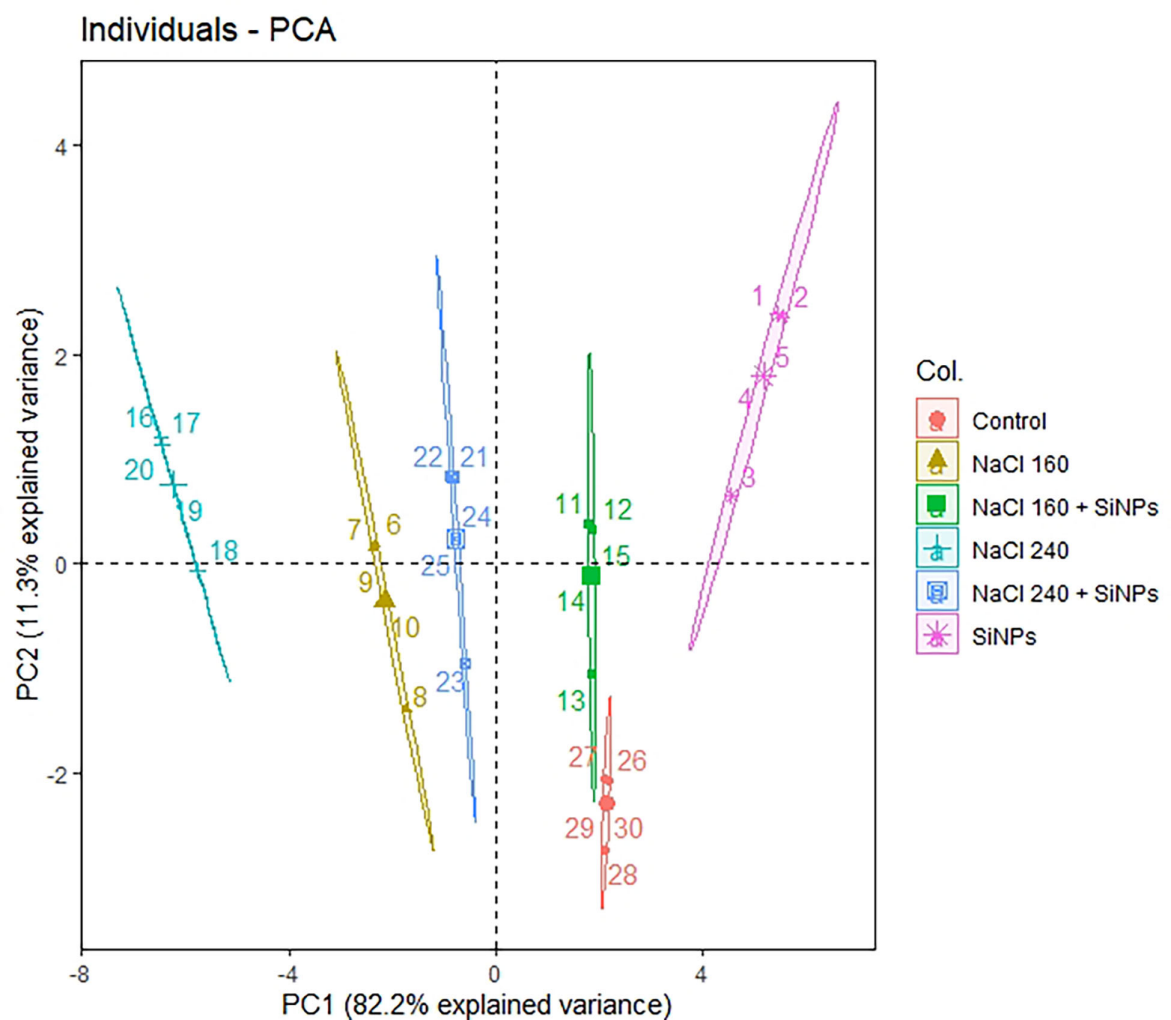
The interconnection among the treatment groups as drawn by PCA scatter plot. Non-overlapping groups are significantly different (α ≤ 0.05) where ellipses hold a 95% confidence level. SiNPs, silicon nanoparticles 150 mg L^-1^; NaCl concentrations are represented in mM.

**Figure 10:**
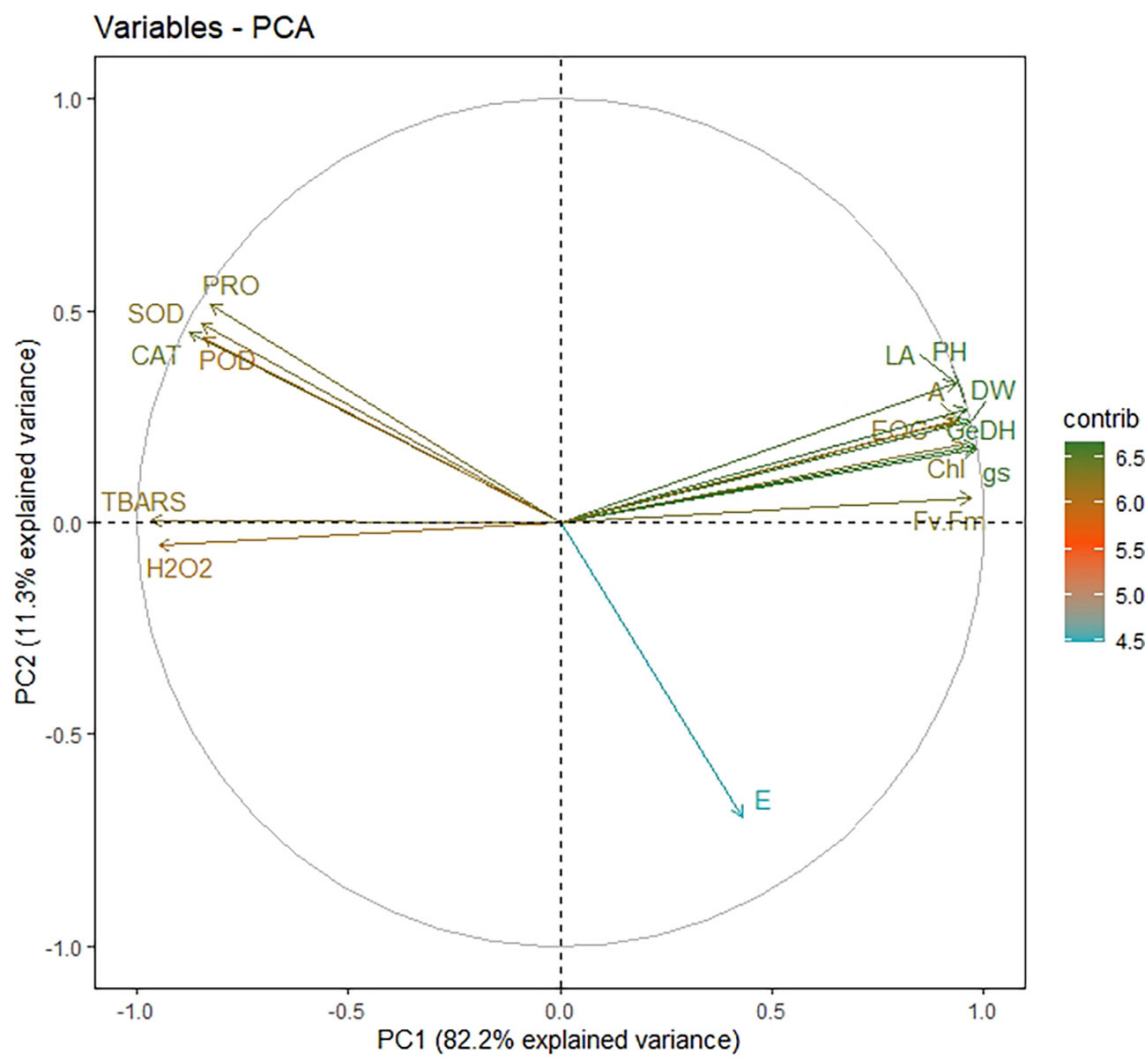
Variable correlation plot depicting the relationship among each variable from different treatment groups. The distance between any two parameters represents their relation intensity. The parameters in the same quadrant are positively related, while the ones from the opposite quadrant are negatively correlated. On the other hand, the distance between the variable and its origin point is directly proportional to the variables’ quality on the factor map. Colour gradients signify each variable’s contribution percentage (contrib) to the principal component. LA, leaf area; GeDH, geraniol dehydrogenase activity; gs, stomatal conductance; Fv/Fm, chlorophyll fluorescence; E, transpiration rate; H_2_O_2_, hydrogen peroxide content; TBARS, thiobarbituric acid reactive substances content; CAT, catalase activity; POD, peroxidase activity; SOD, superoxide dismutase activity; PRO, proline content (overlapped variables: dry weight, plant height, chlorophyll content, photosynthetic CO_2_ assimilation rate, and essential oil content).

**Figure 11:**
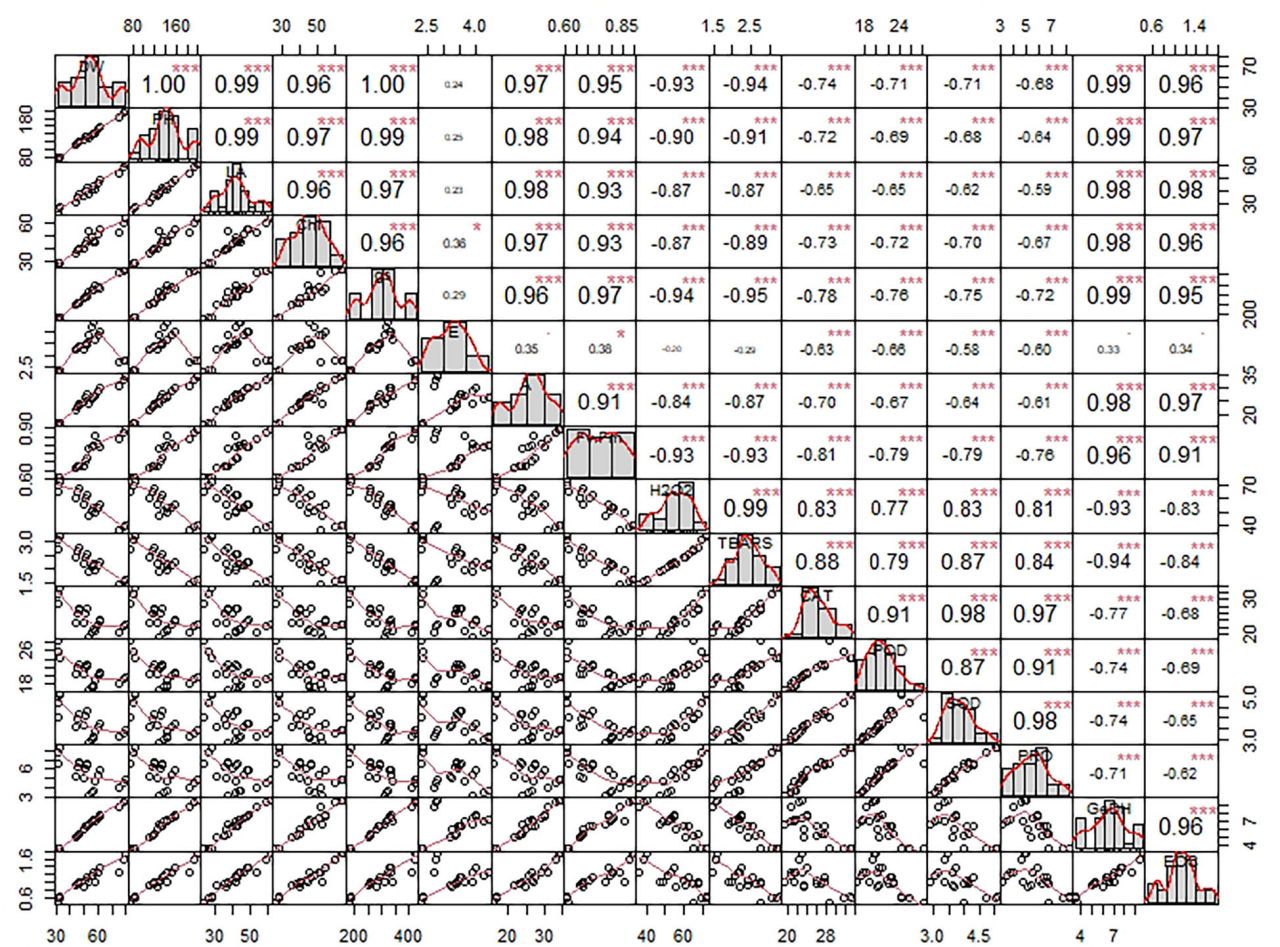
Chart of correlation matrix exploring the intricate relation among variables using Pearson’s method. Each variable is distributed on the chart diagonal. The bottom of the diagonal corresponds to the bivariate scatter plot with a fitted line between any two variables. Further, each box in the diagonal top consists of the correlation values between any two variables and significance levels. Symbols denote significance levels following p-values: . (a = 0.01), * (a = 0.05), *** (a = 0.001). DW, dry weight; PH, plant height; LA, leaf area; CHL, chlorophyll content; gs, stomatal conductance; E, transpiration rate; A, photosynthetic carbon assimilation; Fv/Fm, chlorophyll fluorescence; H2O2, hydrogen peroxide content; TBARS, thiobarbituric acid reactive substances content; CAT, catalase activity; POD, peroxidase activity; SOD, superoxide dismutase activity; PRO, proline content; GeDH, geraniol dehydrogenase activity; EOC, essential oil content.

Principal component analysis (PCA) was done for all plant development and yield variables. We opted for only PC1 and PC2 since they covered about 93% explanation for the total variance during the scree plot analysis (**Supplementary Figure 1**). The scatter plot analysis demonstrated that each treatment group exhibited significantly different responses in lemongrass (**Figure 9**). Plants treated with SiNPs sprays held the highest explained variance with both PC1 and PC2. The same treatment also rendered maximum growth and productivity elicitations in the present study. Contrary to this, the variability of control plants and plants treated with 240 mM NaCl were least explained on PC2 and PC1, respectively. Moreover, all the studied modules exhibited significant interconnection during the PCA variable analysis (**Figure 10**). The variables were further colour-sorted based on their contribution to the principal component. The expected average contribution for each variable to PC1 and PC2 was 6.2% (**Supplementary Figure 2**). Higher values represent a more significant contribution of the variable to PC1 and PC2. The contribution of each variable to the PC1 can be found in **Supplementary Figure 3**. At the same time, variable contribution to the PC2 is depicted in **Supplementary Figure 4**. We also analysed how closely different parameters were related to each other among all treatments. Furthermore, a strong correlation was traced with the correlation matrix chart among all the studied variables (**Figure 11**).

## 4 Discussion

### 4.1 SiNPs positively affect the lemongrass growth and development

Salt extremities (NaCl 160 and 240 mM) restricted plant growth and development in the lemongrass which could be accredited to its moderate salt sensitiveness (Mukarram et al., 2022c). Salinity initiates ionic and oxidative emergency in most plants and restricts the uptake of water and mineral nutrients (Munns, 1993; Hasegawa et al., 2000; Munns, 2002; Ahmad et al., 2022). This induces physiological drought and carbon starvation (Cheeseman, 1988; Kramer and Boyer, 1995; McDowell, 2011; Mukarram et al., 2021c). Nonetheless, SiNPs can act antagonistically to salinity and palliate salt-induced abnormal functionalities in morphological and biochemical modules (Zulfiqar and Ashraf, 2021). It can be ascribed to silicon’s ability to hold various nutrients and water (Savant et al., 1996; Romero-Aranda et al., 2006; Sommer et al., 2006). Further, silicon might promote Na^+^ flow out of the cell through improved H^+^-ATPase activity aiding vacuolar accumulation of Na^+^ and cell compartmentation to increase plant salt tolerance (Yamaguchi et al., 2013). The foliar sprays of 150 mg L^-1^ SiNPs improved plant height, dry weight, and leaf area in the present study as well during both salt regimes. Similar observations were made in *Phaseolus vulgaris* (Alsaeedi et al., 2017), *Ocimum basilicum* (Kalteh et al., 2018), and *Cucumis sativus* (Yassen et al., 2017) with SiNPs application, where it influenced plant metabolism and improved vegetative growth under salinity stress. SiNPs upgraded lemongrass physiology and biochemical status in our case as well. It could be realised by the fact that silicon can escalate the absorption and incarceration of mineral nutrients including nitrogen, phosphorous, potassium, calcium, magnesium, and silicon, and the content of free amino acids, total soluble carbohydrates, and proteins in various plant species (Li et al., 2012; Schaller et al., 2012; Brackhage et al., 2013; Reboredo et al., 2013; Xu et al., 2015; Neu et al., 2017; Xu et al., 2020; Karimian et al., 2021). Further, SiNPs can induce secondary and lateral root growth during salt severity which must have reinforced plant water and nutrient uptake in *Mangifera indica* (El-Dengawy et al., 2021). The positive influence of SiNPs is not limited to dicot plants and taproot systems. Silicon nanoforms seem to support the growth of lateral roots in gymnosperm *Larix olgensis* (Bao-Shan et al., 2004), and both primary and lateral roots in monocot *Brassica juncea* (Pandey et al., 2016), lateral roots in monocot *Musa acuminata* (El-Kady et al., 2017), and lateral root in the fibrous root system of more closely related member of grass family *Oryza sativa* (Isa et al., 2010). Thus, it seems feasible that SiNPs supported fibrous root growth and development in lemongrass plants, promoting water and mineral uptake during saline regimes. Additionally, the magnitude of SiNPs effects seems to vary depending on the application methods such as seed priming (Janmohammadi and Sabaghnia, 2015), root application (Attia and Elhawat, 2021), or foliar application (Mukarram et al., 2021a). Nonetheless, several studies, in concert, suggest SiNPs can enhance growth variables irrespective of applying methods under different environmental conditions in several crops including *Solanum tuberosum* (Mahmoud et al., 2019), *Hordeum vulgare* (Ghorbanpour et al., 2020), *Oryza sativa* (Banerjee et al., 2021), *Saccharum officinarum* (Elsheery et al., 2020), *Coriandrum sativum* (Fatemi et al., 2021), *Triticum aestivum* (Tripathi et al., 2017), and *Polianthes tuberosa* (Karimian et al., 2021).

### 4.2 SiNPs treatment palliate the negative impact of salinity on photosynthesis and stomatal behaviour

Foliar deposition of SiNPs and subsequent non-toxic lignification reduce wilting and retain leaves extended to acquire higher photosynthetic photon flux optimising photosynthesis (Mattson and Leatherwood, 2010; Asgari et al., 2018; Kah et al., 2018). In our case, this could have corresponded to improved chlorophyll fluorescence and net photosynthetic CO_2_ assimilation rate in control and stressed plants with SiNPs application. Etienne et al. (2021) reported that silicon application in *Brassica napus* could regulate the expression level of 296 differentially expressed genes related to photosynthesis, antenna proteins, ribosomes, pentose phosphate pathway, amino acid biosynthesis, and plant hormone signal transduction pathway. This suggests that SiNPs functionality is much more genomic integrated and complex than mere structural support. Many studies indicate that SiNPs can induce several photosynthetic genes belonging to photosystems *(PsaA*, *PsbA*, *PsbW*, *PsbQ*, *PsbP*, and *Psb28*) and electron transport chain (*PetE* and *PetF*) and proteins (Q332S1, Q7F8E8, and P19312) to support photosystem assembly, light-harvesting complex, and thylakoid membrane (Zhang et al., 2018, 2019; Abdelaal et al., 2020; Lesharadevi et al., 2021). The higher chlorophyll content with SiNPs sprays in lemongrass plants suggests that SiNPs favoured the gene expressions of photosynthetic machinery and associated signalling pathways that supported chlorophyll biosynthesis in lemongrass. The higher chlorophyll content along with improved chlorophyll fluorescence could have further supported photosystem biochemistry and palliated salt-induced photosynthetic restrictions in the present study. Similar understandings were developed by Avestan et al. (2019) where SiNPs improved photosynthetic machinery in *Fragaria* × *anansa* plants during salt stress. Siddiqui et al. (2014) argued that SiNPs can reduce the salinity-induced toxic effects in *Cucurbita pepo* by enhancing *A*, g_s_, E, and water use efficiency. SiNPs offered similar effects in our study where salt-stressed lemongrass plants exhibited better g_s_ and E over their non-SiNPs counterparts. Salinity negatively affects stomatal regulation, reducing CO_2_ diffusion and carbohydrate assimilation (Singh and Sharma, 2018). Nonetheless, SiNPs might have intensified carbon accumulation and carboxylation efficiency in lemongrass plants by improving their stomatal activity as was reported in another C_4_ perennial grass *Saccharum officinarum* with silicon (Frazão et al., 2020). We observed an improved photosynthetic assimilation rate with SiNPs in stressed and non-stressed lemongrass plants that can further support this reasoning. Another understanding of silicon-induced stomatal activity comes from hydraulic conductance and aquaporins activity. Dhiman et al. (2021) suggested that silicon increases water uptake and the expression of plasma membrane intrinsic proteins and thus, aquaporins activity under salinity stress. This downplays the repression in root hydraulic conductance and supports stomatal conductivity and water retention during salt stress. SiNPs could perform similar actions in our study with *Cymbopogon flexuosus*. However, further analyses are needed for the elaborative understanding and evidence to support this narrative in lemongrass.

### 4.3 SiNPs stimulate antioxidant metabolism

Higher salt concentrations can disrupt redox homeostasis by overproducing reactive species that compromise the structure and functionality of several biomolecules (Miller et al., 2010; Hossain et al., 2017; Steinhorst and Kudla, 2019). The intricate cross-talk between oxidative species and antioxidants can minimise stress-inflicted damage, thus, perform a cardinal role in stress tolerance (Foyer, 2018; Considine and Foyer, 2021; Mittler et al., 2022). We observed upregulated H_2_O_2_ and TBARS contents during NaCl 160 and 240 mM treatments signifying salinity-induced oxidative bursts in lemongrass plants. This compromised overall plant growth, development, and productivity. Nevertheless, SiNPs were able to diminish both H_2_O_2_ and TBARS contents suggesting an antagonistic mechanism to salinity. It is argued that SiNPs upregulate antioxidant activities for ROS scavenging in stressed cells. Farhangi-Abriz and Torabian (2018); Naguib and Abdalla (2019), and Sharma et al. (2022) concluded that SiNPs can activate the antioxidant system enhancing SOD, CAT, and POD activities to restrict ROS accumulation and associated damage. Data from the present study also suggest a similar feedback mechanism in lemongrass. Several other studies follow this understanding where SiNPs upregulated antioxidative and osmotic defence systems and protected plants against stressful environments (Ashour, 2018; Elsheery et al., 2020; Ghorbanpour et al., 2020; El-Saadony et al., 2021; Alam et al., 2022; Alsamadany et al., 2022). It is suggested that Si-activated isozymes could induce the encoding genes of antioxidant activity under stress (Farouk et al., 2020). Furthermore, SiNPs seem to indulge in gene regulation for PRO biosynthesis thus, having significant osmotic importance during stressful conditions (Siddiqui et al., 2020). Silicon’s interaction with PRO further strengthened the antioxidative system in *Phaseolus vulgaris* during stress (Rady et al., 2019). Mahmoud et al. (2022) reported that SiNPs boosted the PRO and antioxidant contents in *Citrus sinensis* under salt severity. Naguib and Abdalla (2019) primed seeds with SiNPs and observed reduced lipid peroxidation *in Zea mays*. Similarly, SiNPs supplementation brought physiological adjustments to *Cucumis sativus* and palliated salt severity (Yassen et al., 2017).

### 4.4 SiNPs trigger the biosynthesis of lemongrass essential oil

Essential oil productivity has a dual relationship with stressful environments. While milder stress induces essential oil content, severe stress conditions can cause a considerable reduction in overall oil yield (Mirzaei et al., 2020; Mukarram et al., 2022c). The plummet in LEO content under 160 and 240 mM NaCl could result from poor plant growth and development owing to ionic, osmotic, and oxidative imbalance and retarded plant-water relation, nutrient uptake, photosynthates production, and source-sink potential (Idrees et al., 2012; Dagar et al., 2013; Shahid et al., 2020). However, SiNPs sprays improved essential oil productivity in lemongrass plants during both salt intensities. This can be ascribed to silicon’s positive effect on water and nutrient uptake and source-sink potential in grass and legume plant functional types (Xu et al., 2020) or the SiNPs constructive role in photosynthesis, stomatal conductance, and ROS and antioxidant metabolism as we observed in our previous study with *Cymbopogon flexuosus* plants (Mukarram et al., 2021a). Furthermore, SiNPs upregulate GeDH activity which is believed to play a cardinal role in oil enrichment in lemongrass (Mukarram et al., 2021a). Kalteh et al. (2018) and Alsaeedi et al. (2019) observed similar results where SiNPs improved yield components in *Ocimum basilicum* and *Cucumis sativus*, respectively. Our finding agrees with a previous study with *Oryza sativa* where SiNPs application delivered higher crop production during salinity stress (Badawy et al., 2021). The obtained results of higher lemongrass yield with SiNPs find further support from several other studies where SiNPs exhibited similar effects in *Solanum lycopersicum* (Haghighi and Pessarakli, 2013), *Solanum tuberosum* (Kafi et al., 2021), *Triticum aestivum* (Mushtaq et al., 2017), and *Musa acuminata* (Mahmoud et al., 2020) under salt stress.

## 5 Conclusion

**Figure 12:**
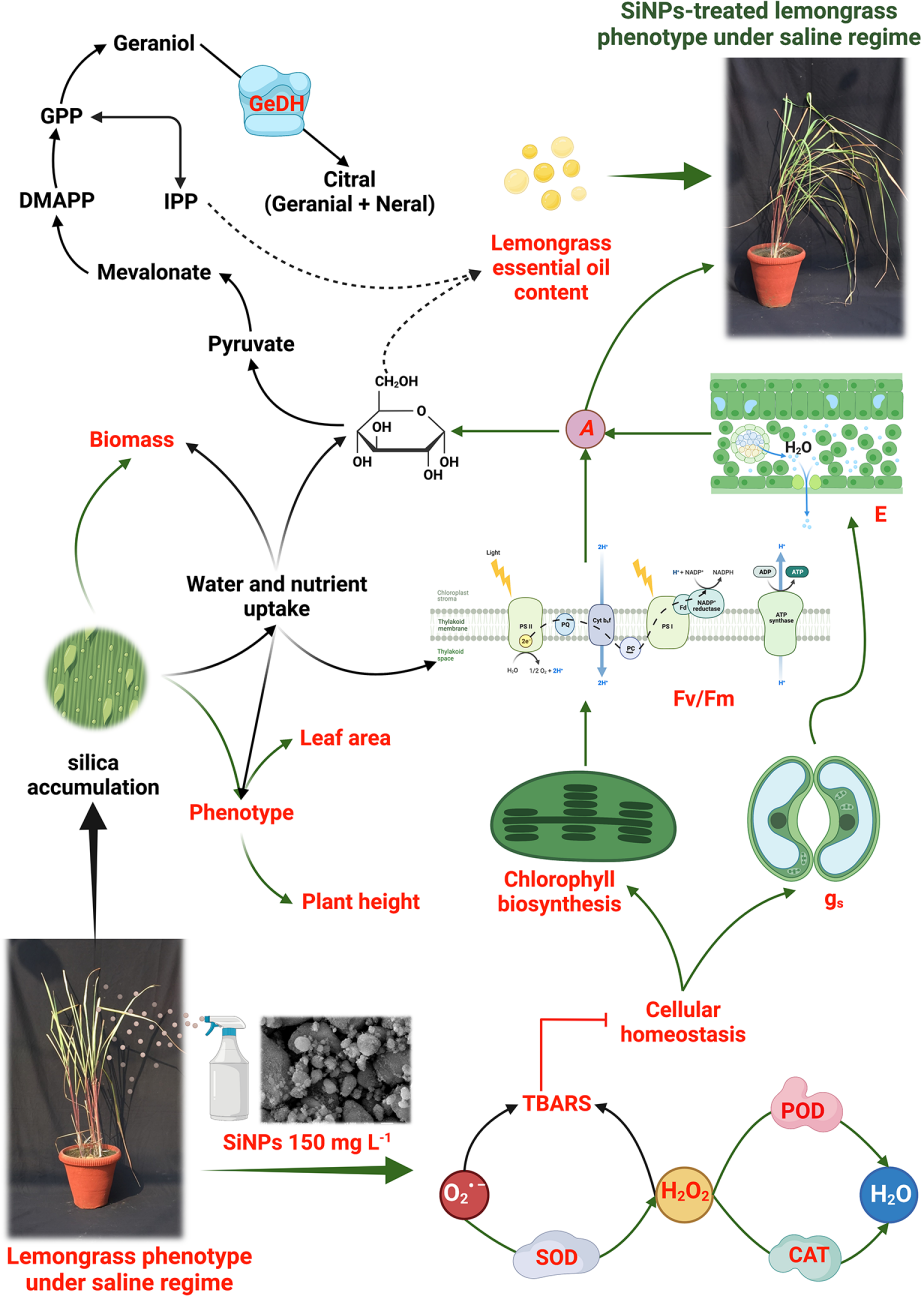
Proposed *modus operandi* of silicon nanoparticles (SiNPs) in lemongrass as was developed during the present study. Our results suggest that SiNPs palliate salt-induced oxidative stress by boosting antioxidant metabolism (such as SOD, CAT, and POD). Improved cellular homeostasis could support chlorophyll biosynthesis and PSII efficiency (Fv/Fm). Subsequent upgradation in stomatal dynamics (such as g_s_ and E) would assist lemongrass with a higher photosynthetic CO_2_ assimilation rate (*A*). Further, a higher *A* is expected to generate more glucose which can undergo a mevalonate or mevalonate-independent pathway to confer improved essential oil productivity in salt-stressed lemongrass. The overall upgradation of plant physiology coupled with improved water and nutrient uptake during salt stress can render morphological improvements in lemongrass such as dry weight, leaf area, and plant height. The studied phenomena are coloured in red while the green arrows show SiNPs-induced elicitation of the process.

The obtained results support the effectiveness of SiNPs-induced protection in lemongrass against salt stress (NaCl 160 and 240 mM). We found that SiNPs redress photosynthetic performance and stomatal conductance in salt-stressed lemongrass. At the same time, foliar sprays of SiNPs instigated antioxidant production to defend lemongrass plants against salt-induced oxidative stress. Upregulated SOD, CAT, POD, and PRO facilitated cellular homeostasis in the lemongrass. Thus, we noticed reduced H_2_O_2_ and TBARS content in NaCl 160 and 240 mM treated plants. Further, SiNPs boosted GeDH enzyme activity and essential oil content in both saline regimes. SiNPs could downright redress NaCl 160 mM-induced damages. However, NaCl 240 mM conferred irreversible damage to the lemongrass growth, development, and productivity modules. A working model for these coordinated biochemical effects is proposed in **Figure 12** which is based on our understanding developed during the present study and the insights from our previous studies with lemongrass (see reference list for details). In summary, our results indicate that SiNPs application upgrades plant physiology and triggers cellular defence of lemongrass plants against high salt stress (≤240 mM). These, in concert, brought improved crop productivity in the present study. Therefore, it is proposed that SiNPs could be a useful biotechnological tool to palliate salinity stress in lemongrass crops and that its use could be extrapolated to other agricultural species. On this note, future trends could include the followings:

1. It would be remarkable to know how lemongrass would respond to a mixture of several stresses such as drought, salinity, and high temperature with external SiNPs application.
2. It is high time to know if SiNPs can induce heat-shock proteins or vacuolar H^+^-ATPase channels in plants under global climate change scenarios.
3. What individual genes can SiNPs directly target to induce photosynthesis and antioxidant metabolism?
4. Do SiNPs induce Na^+^ exclusion at soil-root interphase in saline soil settings?
5. Could the increase in essential oil in stressful situations be considered a reliable marker of stress?

## Data availability statement

The original contributions presented in the study are included in the article/**Supplementary Material**. Further inquiries can be directed to the corresponding author.

## Author contributions

MM: Data curation, Formal analysis, Funding acquisition, Investigation, Writing – Original draft preparation. MK: Conceptualization, Funding acquisition, Methodology, Project administration, Resources, Supervision, Validation, Visualization, Writing – Review & Editing. DK: Validation, Visualization, Writing – Review & Editing. AL: Validation, Visualization, Writing – Review & Editing. FC: Validation, Visualization, Writing – Review & Editing. All authors contributed to the article and approved the submitted version.
