# Supplementary figures and images for: Silicon nanoparticles (SiNPs) restore photosynthesis and essential oil content by upgrading enzymatic antioxidant metabolism in lemongrass (Cymbopogon flexuosus) under salt stress
Source: Front Plant Sci. 2023 Feb 17;14:1116769. doi: 10.3389/fpls.2023.1116769 (PMC9981966; doi:10.3389/fpls.2023.1116769)

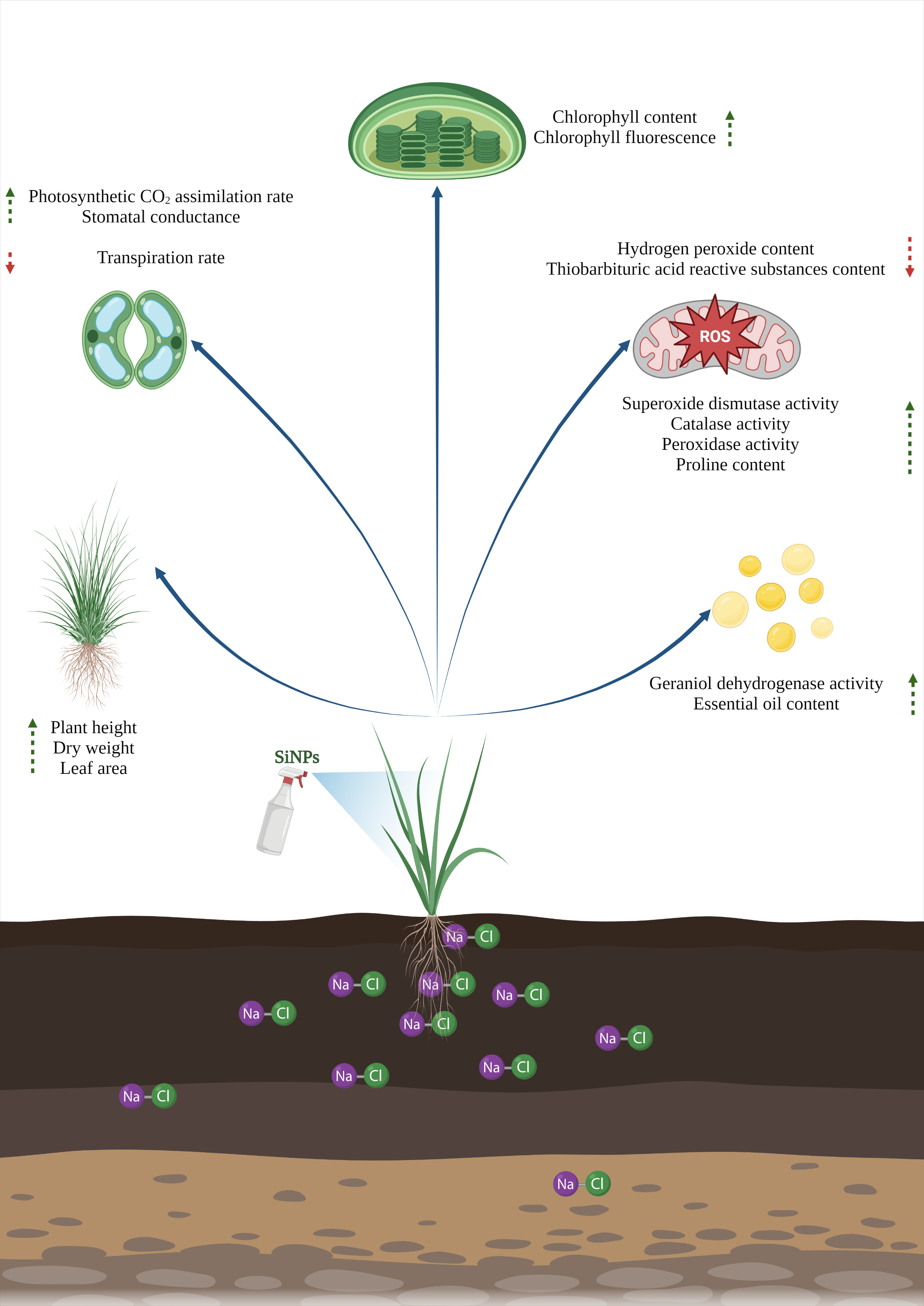

Supplement: Supplementary file 1 [file Image_1.jpeg]
