# Supplementary material for: Silicon nanoparticles (SiNPs) restore photosynthesis and essential oil content by upgrading enzymatic antioxidant metabolism in lemongrass (Cymbopogon flexuosus) under salt stress
Source: Front Plant Sci. 2023 Feb 17;14:1116769. doi: 10.3389/fpls.2023.1116769 (PMC9981966; doi:10.3389/fpls.2023.1116769)
Supplement: Supplementary file 2 [file Table_1.docx]

**Silicon nanoparticles (SiNPs) restore photosynthesis and essential oil content by upgrading enzymatic antioxidant metabolism in lemongrass (*Cymbopogon flexuosus*) under salt stress**

Mohammad Mukarram^1,2,*^, M. Masroor A. Khan^1^, Daniel Kurjak^3^, Alexander Lux^4,5^, Francisco J Corpas^6^

^1^Advance Plant Physiology Section, Department of Botany, Aligarh Muslim University, Aligarh 202002, India

^2^Department of Phytology, Faculty of Forestry, Technical University in Zvolen, T. G. Masaryka 24, 96001, Zvolen, Slovakia

^3^Department of Integrated Forest and Landscape Protection, Faculty of Forestry, Technical University in Zvolen, T. G. Masaryka 24, 96001, Zvolen, Slovakia

^4^Department of Plant Physiology, Faculty of Natural Sciences, Comenius University in Bratislava, Ilkovicova 6, Bratislava, Slovakia

^5^Institute of Chemistry, Slovak Academy of Sciences, Dúbravská cesta 9, Bratislava, Slovakia

^6^Department of Stress, Development and Signaling in Plants, Antioxidant, Free Radical and Nitric Oxide in Biotechnology, Food and Agriculture Group, Estación Experimental del Zaidín, Consejo Superior de Investigaciones Científicas (CSIC), Granada, Spain

***Correspondence:** Mohammad Mukarram ([mdmukarram007@gmail.com](mailto:mdmukarram007@gmail.com))

**ORCID:** Mohammad Mukarram (0000-0002-9034-9366), M. Masroor A. Khan (0000-0002-4530-9082), Daniel Kurjak (0000-0002-2489-8463), Alexander Lux (0000-0001-8651-8166), Francisco J Corpas (0000-0002-1814-9212)

^
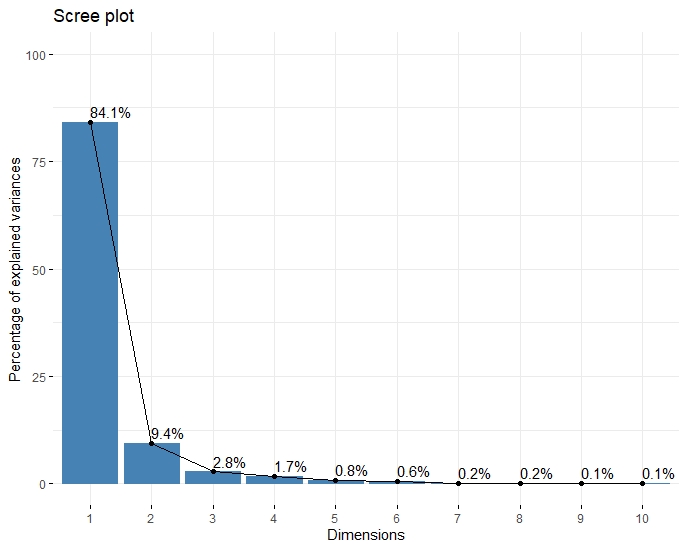
^

**Supplementary Fig.1** Scree plot displaying the first two principal components (PC1 and PC2) capturing the most variation, i.e., about 93%.


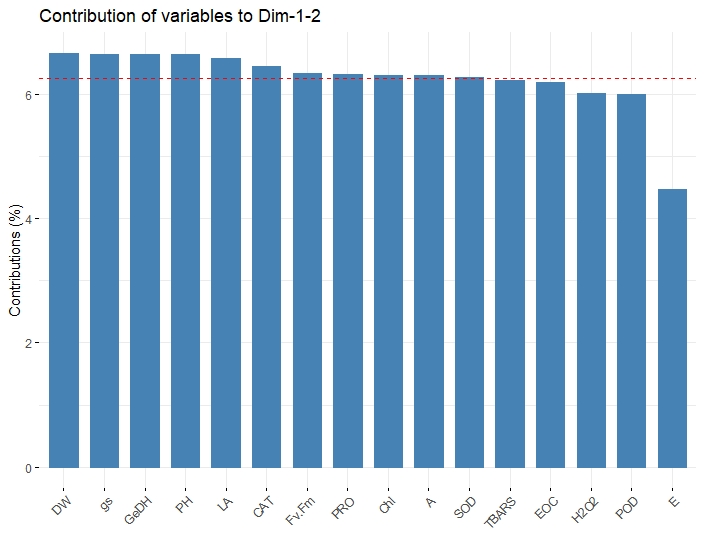


**Supplementary Fig. 2** Contribution percentage of each variable to both principal components
i.e., PC1 and PC2 where the first seven variables exhibited higher contribution percentages than the average (6.2%).). The broken red line points to each variable's expected average contribution percentage to PC1 and PC2. DW, dry weight; PH, plant height; LA, leaf area; CHL, chlorophyll content; gs, stomatal conductance; E, transpiration rate; *A*, photosynthetic carbon assimilation; Fv/Fm, chlorophyll fluorescence; H_2_O_2_, hydrogen peroxide content; TBARS, thiobarbituric acid reactive substances content; CAT, catalase activity; POD, peroxidase activity; SOD, superoxide dismutase activity; PRO, proline content; GeDH, geraniol dehydrogenase activity; EOC, essential oil content.


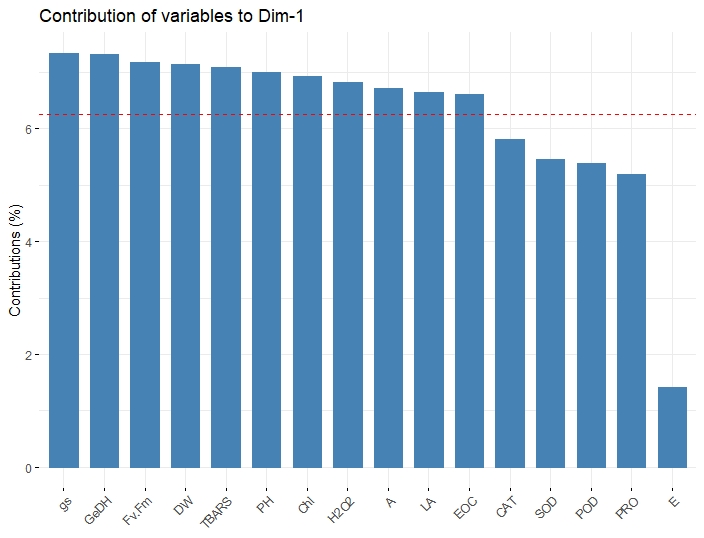


**Supplementary Fig. 3** Contribution percentage of each variable to the first principal component (PC1). The broken red line points to each variable's expected average contribution percentage to the PC1. DW, dry weight; PH, plant height; LA, leaf area; CHL, chlorophyll content; gs, stomatal conductance; E, transpiration rate; *A*, photosynthetic carbon assimilation; Fv/Fm, chlorophyll fluorescence; H_2_O_2_, hydrogen peroxide content; TBARS, thiobarbituric acid reactive substances content; CAT, catalase activity; POD, peroxidase activity; SOD, superoxide dismutase activity; PRO, proline content; GeDH, geraniol dehydrogenase activity; EOC, essential oil content.

**
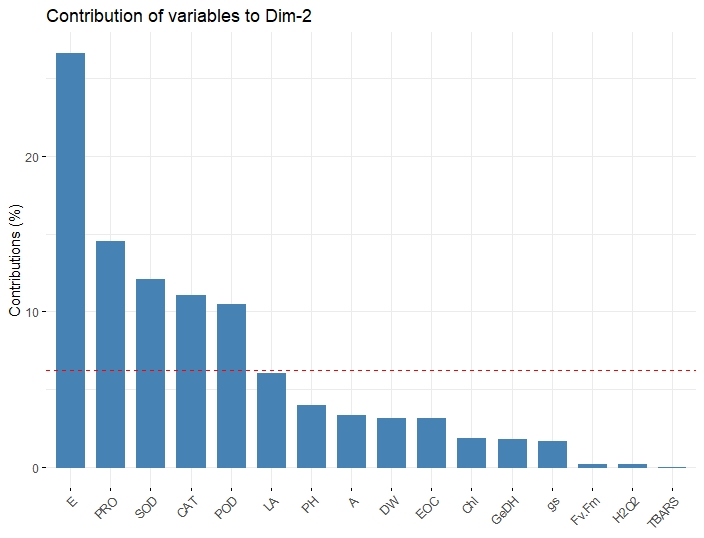
**

**Supplementary Fig. 4** Contribution percentage of each variable to the second principal component (PC2). The broken red line points to each variable's expected average contribution percentage to the PC2. Contrary to PC1, transpiration rate (E) contributed the highest share to PC2. DW, dry weight; PH, plant height; LA, leaf area; CHL, chlorophyll content; gs, stomatal conductance; E, transpiration rate; *A*, photosynthetic carbon assimilation; Fv/Fm, chlorophyll fluorescence; H_2_O_2_, hydrogen peroxide content; TBARS, thiobarbituric acid reactive substances content; CAT, catalase activity; POD, peroxidase activity; SOD, superoxide dismutase activity; PRO, proline content; GeDH, geraniol dehydrogenase activity; EOC, essential oil content.
